# Study on the Effect of PTFE/Cu Composite Material Preparation Process on Penetration Performance

**DOI:** 10.3390/polym15173504

**Published:** 2023-08-22

**Authors:** Jianya Yi, Ruijie Hao, Xuezhi Tang, Siman Guan, Zhijun Wang, Jianping Yin

**Affiliations:** 1 School of Mechatronic Engineering, North University of China, Taiyuan 030051, China; hrj2021315@163.com (R.H.); gsm2021315@163.com (S.G.); wzj@nuc.edu.cn (Z.W.); yjp123@nuc.edu.cn (J.Y.); 2 Chongqing Hongyu Precision Industrial Co., Ltd., Chongqing 402760, China; ha_txz@126.com

**Keywords:** PTFE/Cu composite material, preparation process, liner, penetration performance, numerical simulation

## Abstract

The jet formed by the traditional metal liner has a slender shape. The diameter of the jet head is consistent with that of the tail, and the ductility is good. When it is used to penetrate the target, it has a good damage effect. The low-density jet formed by the PTFE/Cu liner, according to the different preparation processes and densities, has different degrees of radial expansion. This phenomenon may lead to the expansion of the jet head during the penetration process, resulting in a damage effect, which is different from the previous jet on the target. In this paper, the numerical simulation of PTFE/Cu liners with different preparation processes penetrating steel targets is carried out, and the effects of different preparation processes and liner density on the penetration characteristics of jets penetrating steel targets are compared and analyzed. The PTFE/Cu shaped charge liner was processed according to different preparation processes, and the jet penetration steel target experiment was carried out, so as to verify and analyze the numerical simulation results.

## 1. Introduction

With the wide application of shaped charge structure technology in the military field and the continuous improvement of modern armor protection technology, people have put forward higher requirements for the performance of shaped charge warheads. In addition to achieving a larger penetration depth, it is also necessary to achieve a large penetration aperture. In this context, metal/polymer composite liners came into being.

The research on metal and polymer composite shaped charge liners is mainly divided into two types. One is the low-density active composite shaped charge liner. For the metal/non-metal active material shaped charge liner, it not only has the original penetration effect, but also has combustion, detonation, heat release, and other after effects, which further strengthen the damage mode of anti-armor ammunition to the target. The other is the low-density inert composite liner. At present, the research on the low-density inert composite liner is focused on the mutual compensation of the mechanical properties between metal and non-metal, so as to maximize the use of the advantages of the two materials. Adding different fillers to the PTFE matrix can effectively improve the mechanical properties of the material and make it exhibit better physical properties than a single material [1,2,3]. At present, most of the research on metal/non-metal liners is about PTFE-based or PTFE/Al-based active energetic materials. With the reactive materials becoming more and more popular, many scholars have carried out a series of studies on the active materials of PTFE matrix, including its formulation [4,5], mechanical properties [6], chemical reaction energy release [7], and damage mechanism [8]. The research on PTFE/Cu composites is also gradually developing, including the influence of the shape, content, and size of copper powder on the tribological properties of composites [9], crack propagation mechanism [10], and so on.

The jet formed by the PTFE composite material as the liner has better performance than the traditional metal jet in some aspects when penetrating the target. The jet formed by the Al/PTFE reactive material liner prepared by cold-pressing sintering has a larger aperture and a lower penetration depth for the penetration of thick steel plates compared with the traditional metal liner shaped charge jet [11]. Compared with the traditional metal liner, the reactive material liner has a stronger penetration after effect, so it is gradually becoming more commonly used as the liner material [12]. Yi et al. [13] studied the jet formed by PTFE liner and pure copper liner, and found that the polymer expansion jet has a larger penetration aperture than the typical copper jet penetration performance. Hirsch and Sadwin [14] pointed out that a cavity with increased diameter will be formed near the bottom of the penetration hole of the jet formed by the shaped charge liner made of highly compressible materials (such as thermoplastics), which will lead to the failure of brittle materials such as concrete.

In this paper, three kinds of PTFE/Cu material liner preparation processes were proposed. Through the combination of numerical simulation and experimental verification, the penetration performance of PTFE/Cu jets with different preparation processes was studied. The effects of liner density, preparation process, and sintering sequence on the damage performance of PTFE/Cu jet against the steel target were investigated. In our study, different processes were used to prepare the liner, which has never appeared in the previous literature. Our research lays a foundation for the study of the reaming effect of polymer/metal composite jets on targets.

## 2. Materials

PTFE powder is insoluble in any solvent and is not easily corroded by other substances. Its surface tension is the smallest in solid materials, and it does not adhere to any substance. It has excellent chemical stability and good mechanical toughness at low temperature. The molding method is mainly divided into two kinds, one of which is to use PTFE resin to form products directly. The molding process includes molding, hydraulic, pushing, extrusion, and spraying. The other method is mainly to process PTFE plastic sheets and thin strips. In this paper, PTFE/Cu composite liners with different mass ratios were prepared by extrusion molding, molding sintering, and hot-pressing sintering. The molding sintering temperature is not more than 380 °C.

### 2.1. Hot-Pressing Sintering

Hot-pressing sintering refers to the process of filling the dry mixed powder into the model and heating it through heat conduction or heat radiation. At the same time, the mold is subjected to one-way or two-way pressure to make the molding and sintering complete simultaneously.

**Figure 1:**
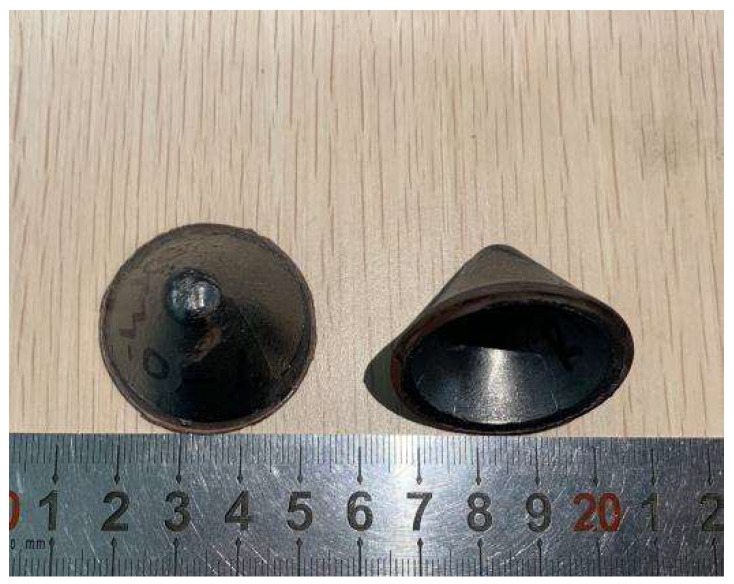
Hot-pressing sintering liner.

Considering the poor heat transfer performance of PTFE, its crystallization transformation point is 327 °C, and then it becomes an amorphous gel state upwards of this. The melt viscosity is high and it is not easy to flow, so its sintering temperature needs to be higher than 327 °C. At the same time, in order to improve the quality of the obtained specimens, it is necessary to pay attention to the heating rate during sintering. The whole hot-pressing sintering process is completed under the protection of argon atmosphere. The temperature control degree control panel is used to control the temperature of the sintering process of the specimen. In order to improve the stability of the temperature change and make the actual temperature closer to the calculated temperature, the heating stage is carried out by variable rate heating, that is, the second heating rate is lower than the first heating rate, and then the heat preservation and cooling are carried out. As shown in Figure 1, it is a shaped charge liner prepared by hot-pressing sintering.

### 2.2. Molded Sintering

Molded sintering is to cold-press the powdered material into a dense preform of various shapes, and then heat the preform to a temperature higher than its crystalline melting point, so that the particles dissolve each other to form a dense continuous whole, and finally cool to room temperature to obtain the product.

**Figure 2:**
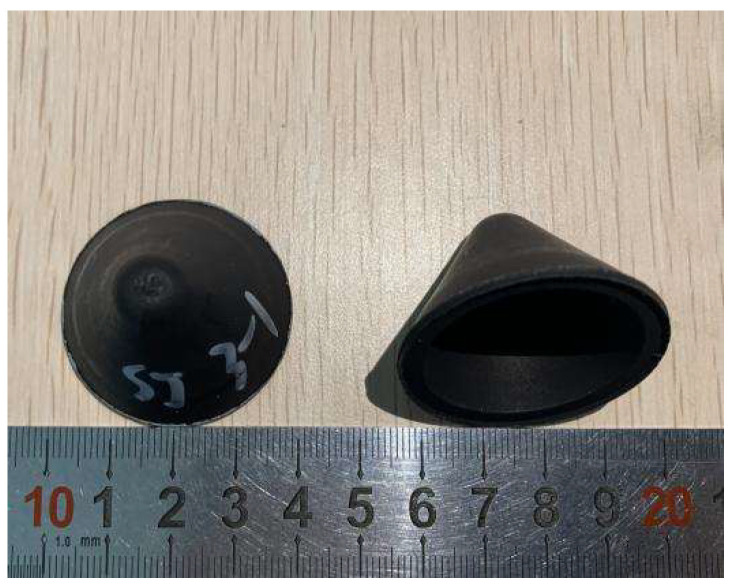
Molded sintering liner.

In this paper, PTFE/Cu composites were sintered by high temperature sintering furnace. Figure 2 shows the prepared molded sintering liner.

### 2.3. Extrusion Molding

**Figure 3:**
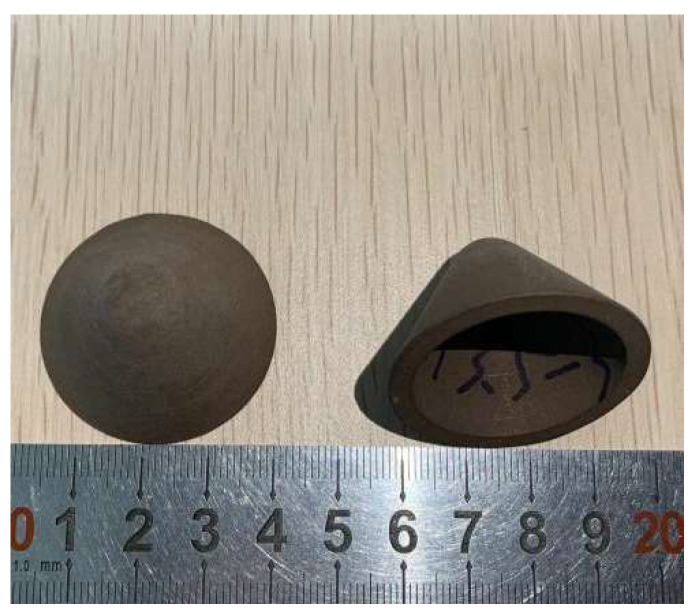
Extrusion molding liner.

Extrusion molding method refers to a kind of processing and preparation method in which plastic materials form products from the hole mold under the action of a strong external force. Based on this preparation technology, products with a length much larger than the interface can be prepared. Figure 3 is the liner produced by extrusion molding process.

## 3. Theoretical Analysis of PTFE/Cu Jet Reaming

When penetrating a medium-thick target, the shaped charge jet simultaneously performs axial penetration and radial hole expansion of the target. The theory of axial penetration can be roughly divided into an incompressible model and a compressible model. The main difference between the two is that the latter considers the compressibility of the projectile/target material. Predecessors have proposed a sufficiently complete model to be applied to the analysis of axial penetration. For aluminum targets, the most reasonable model is the compressible model derived by Fils using the quadratic Mei–Grüneisen state equation.

Based on the two conclusions of Szendrei [15] and the compressible model of Flis [16,17], a compressible model of radial reaming is derived. This model derives two conclusions from Szendrei: the axial pressure of jet penetration affects the initial radial pressure of jet reaming; the relationship between the stagnation pressure of jet reaming and the velocity of target reaming is analogous to the relationship between the stagnation pressure of jet penetration and the velocity of jet penetration. In view of the fact that Flis’s compressible model takes into account the deformation of the material during penetration and reaming, the internal energy and pressure changes of the material, and increases the analysis of the process of the material from the shock wave interface to the projectile/target interface, a new reaming equation is derived on the basis of it [18].

**Figure 4:**
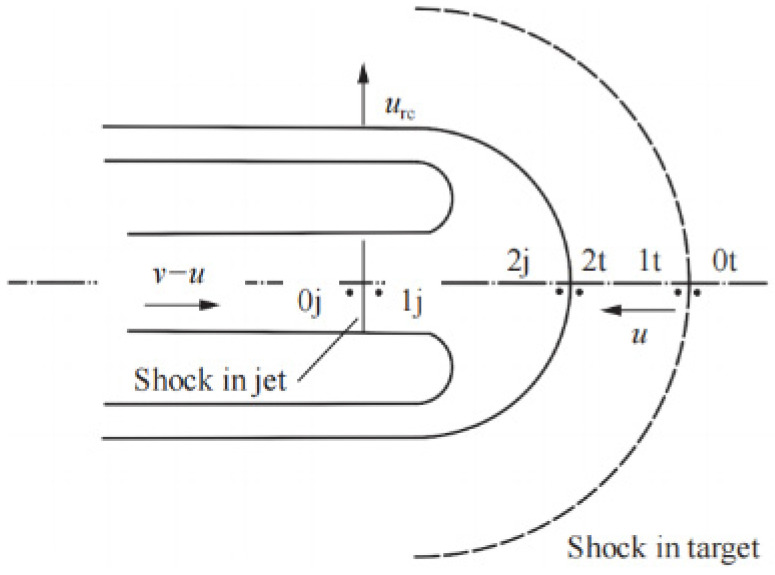
Penetration velocity and reaming velocity distribution and flow field in the process of penetration [18].

The schematic diagram of the two interfaces is shown in Figure 4. The subscript in front of the shock wave interface is state 0, that is, the initial state. The subscript after the interface is state 1, and the subscript at the projectile/target interface is state 2; j represents the jet, t represents the target, v is the velocity of the shaped jet, and u is the penetration velocity. The incompressible model is directly from state 0 to state 2, while the compressible model of Flis considers the process from state 0 to state 1 on this basis, and assumes that the internal energy change from state 1 to state 2 is isentropic.

Assuming that the product of pressure and area is a constant for the relationship between the reaming stagnation pressure *p* and the stagnation pressure *p*_2*t*_ at the corresponding velocity, the relationship between the stagnation pressure *p* and the stagnation pressure *p*_2*t*_ at this velocity is:

(1) $$p=\frac{r_{j}^{2}}{r^{2}}p_{2t}$$

In the formula: *r_j_* is the jet radius, *r* is the reaming radius.

In the compressible Bernoulli equation, the stagnation point pressure *p*_2*t*_ is the sum of the dynamic pressure and the static pressure at the stagnation point, that is:

(2) $$p_{2t}=k_{t}\frac{1}{2}p_{0t}u^{2}-k_{t}p_{0t}\left(E_{2t}-E_{0t}\right)+R_{t}$$

In the formula: $k_{t}\frac{1}{2}p_{0t}u^{2}$ is the dynamic pressure term of the penetration velocity *u*, $k_{t}=\rho_{2t}/\rho_{0t}$ is the ratio of the target density *ρ*_2*t*_ and the initial density *ρ*_0*t*_ at the projectile/target interface; *E*_2*t*_ is the internal energy of the target at the interface between the projectile and the target, and *E*_0*t*_ is the initial internal energy of the target. The strength *R_t_* of the material after the static pressure is the wavefront is compared with the stagnation pressure of the incompressible model. The compressible model considers the change of material density and internal energy by increasing *k_t_* and the internal energy term $k_{t}p_{0t}\left(E_{2t}-E_{0t}\right)$. However, the internal energy term has little effect on the penetration, so it can be considered that it has little effect on the reaming process, and the item is ignored. As shown in Figure 4, the direction of the expansion velocity *u_rc_* is perpendicular to the penetration velocity *u*. According to Szendrei’s second argument, the relationship between the stagnation pressure *p* and the expansion velocity *u_rc_* is obtained from Equation (2):

(3) $$p=k_{t}\frac{1}{2}p_{0t}u^{2}+R_{t}$$

From Equations (1) and (3), it can be obtained that the reaming velocity of a certain aperture is:

(4) $$u_{rc}=\sqrt{\frac{A}{r^{2}}-B}$$

In the formula: $A=\frac{2r_{j}^{2}p_{2t}}{\rho_{2t}}$ is the stagnation point pressure term, and $B=\frac{2R_{2t}}{\rho_{2t}}$ is the target strength term.

The relationship between the reaming radius *r* and the time *t* can be expressed as:

d*r* = *u_rc_*d*t* (5)

The simultaneous Equations (4) and (5) and integral, can be obtained:

(6) $$r=\sqrt{\frac{A}{B}-{\left(\sqrt{\frac{A}{B}-r_{j}^{2}}-t\sqrt{B}\right)}^{2}}$$

According to the combined Equations (4) and (6), the jet radius, stagnation point pressure, target strength, and target density at the stagnation point are the main factors affecting the reaming of shaped charge jet.

It can be seen from Equation (4) that when the reaming velocity *u_rc_* = 0, it means that the reaming reaches the maximum radius *r_max_*, then:

(7) $$r_{max}=\sqrt{\frac{A}{B}}=r_{j}\sqrt{\frac{p_{2t}}{R_{t}}}$$

Based on the compressible model of jet penetration and Szendrei’s two judgements, a reaming model of shaped charge jet considering the compressibility of materials is obtained. Compared with the previous models, the model in this paper considers the compressibility of the projectile/target material, and can predict the hole expansion of the target more accurately, especially the high compressibility target.

## 4. Experimental Methods

### 4.1. Numerical Simulation of PTFE/Cu Jet Penetrating Steel Target

In order to better analyze the characteristics of the PTFE/Cu jet penetrating steel targets with different preparation processes, numerical simulations of different PTFE/Cu shaped charge liners were carried out.

The explosive charge structure is a stern type, the stern width is 22 mm, the angle is 2θ = 24°, the shell-less charge is adopted, and the thickness of the liner is equal, which is the arc and cone combined liner. Among them, the radius of the cone top of the liner is 6 mm, the wall thickness is δ = 3 mm, the cone angle of the liner is 2*α* = 60°, the height of the liner is l = 26.04 mm, the diameter of the charge is D_k_ = 37 mm, and the height of the charge is H = 47 mm.

**Figure 5:**
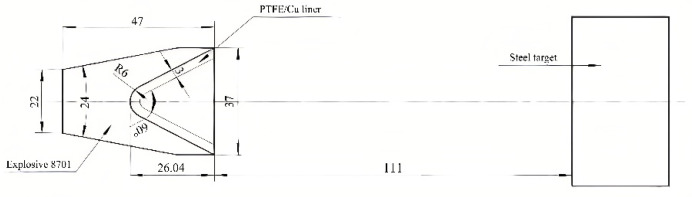
Structural diagram of shaped charge and target plate.

According to the previous scholars’ research on PTFE and modified PTFE penetrating target plate, the best stand-off of penetration is 3D, that is, three times the charge diameter. Therefore, in this section, the burst height of PTFE/Cu jet penetrating target plate is determined to be 3D_k_, that is, 111 mm. The structure diagram of shaped charge and target plate is shown in Figure 5.

**Figure 6:**
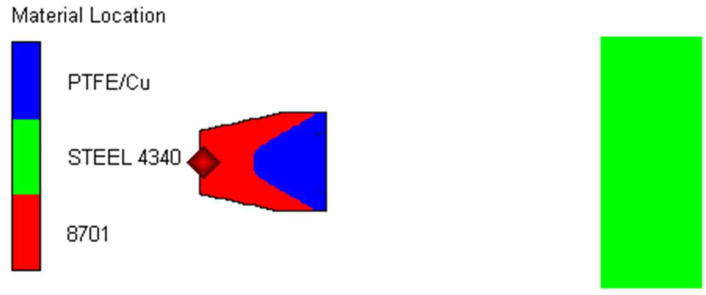
Finite element model.

In order to study the penetration effect of the jet more intuitively and effectively, the simulation is based on AUTODYN software 19.2, and 3D modeling is adopted. As the forming and penetration of jet are symmetrical, 1/4 modeling is carried out, and the finite element model is shown in Figure 6. Our finite element simulation uses the SPH algorithm, and there is no boundary problem. The gap of SPH particles is 0.05 cm, and the mesh size of the target plate is 0.05 cm.

The constitutive model of PTFE/Cu material is Johnson–Cook, and the equation of state is shock. In this paper, the constitutive equations of all PTFE/Cu materials are fitted by ourselves. The dynamic and static mechanical properties of the material samples are tested at room temperature. The constitutive equations of PTFE/Cu materials at room temperature are fitted without considering the influence of temperature softening effect. The specific parameters are shown in Table 1.

Explosive 8701 is selected as the explosive, and the JWL state equation is used to describe the detonation process of explosive. The specific parameters are shown in Table 2.

In the table: *ρ* is the average density of the main charge prepared for the experiment, *D* is the detonation velocity, *E* is the energy density per unit volume, and *P_CJ_* is the detonation pressure of the explosive.

The target plate is made of 4340 steel, and its material model is Johnson–Cook. The main parameter settings are shown in Table 3.

### 4.2. Experiment Verification of Penetrating Steel Target

In order to verify the damage efficiency of the jet penetrating the target plate, the penetration experiments of PTFE/Cu liners with different processes into steel targets were carried out, and the penetration performance of PTFE/Cu jets under different working conditions was compared and analyzed with the numerical simulation results. Considering the experiment cost, the verification experiment of penetrating steel target was only carried out on parts of the liners.

The verification experiment is a total of six sets, and the liner and related experiment data are shown in Table 4. In order to measure the penetration velocity of the jet, two sets of experiment conditions with a stand-off of 2D were set up.

**Figure 7:**
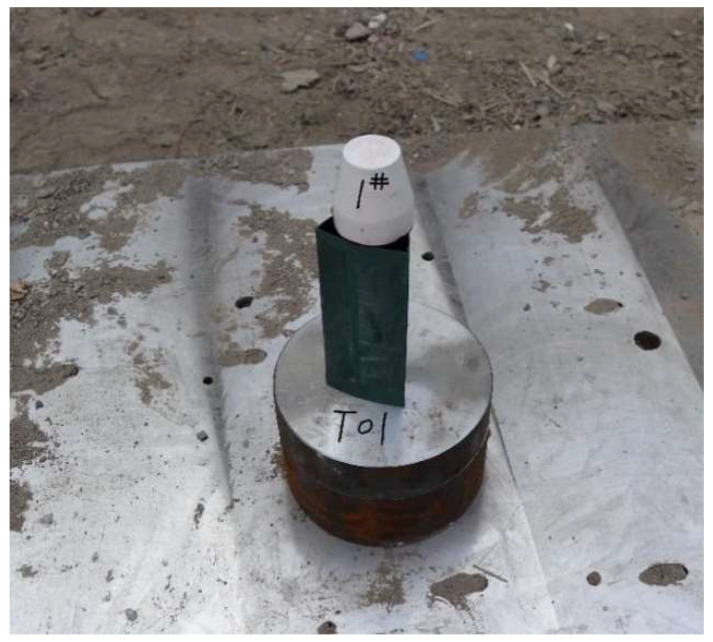
Setting of penetration experiment.

The experiment site is arranged as shown in Figure 7, in which the stand-off barrel is made of highland barley paper, which does not affect the transmission of detonation wave during jet forming. The steel target is 4340 steel with a diameter of 120 mm and a height of 30 mm.

## 5. Results and Discussion

### 5.1. Numerical Simulation Results

#### 5.1.1. Effect of Liner Density on Jet Penetration Characteristics

In order to study the effect of different liner density on jet penetration performance, two sets of liners were selected, which were hot-pressing sintering liners with density of 3 g/cm^3^, 3.5 g/cm^3^, and 4 g/cm^3^ and molded sintering liners with the same density. The penetration results of steel targets were compared and analyzed.

**Figure 8:**
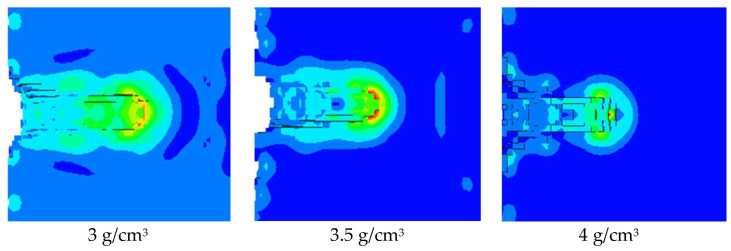
Damage effect of hot-pressing sintering PTFE/Cu jet on steel target.

**Figure 9:**
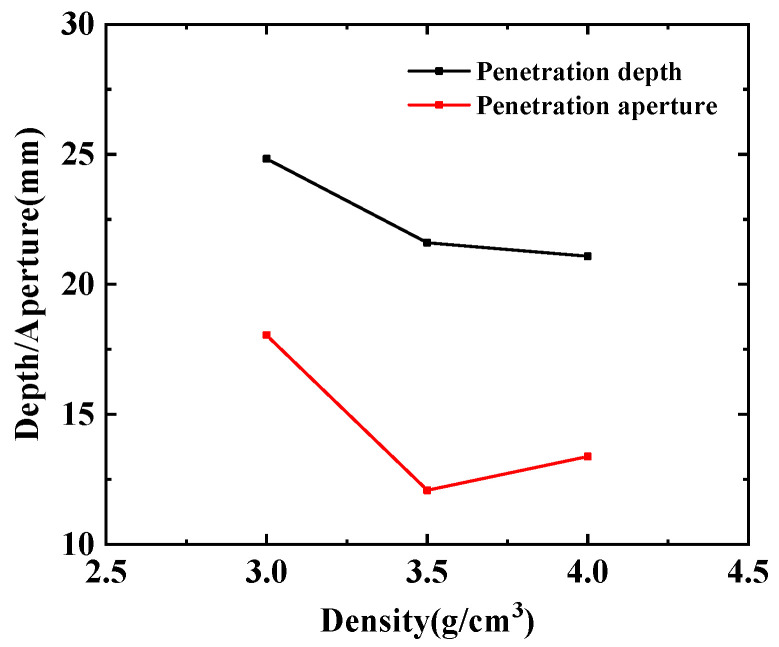
Penetration characteristics of hot-pressing sintered PTFE/Cu jet on steel target.

As shown in Figure 8, the pressure nephogram of the jet formed by the hot-pressing sintering PTFE/Cu liner penetrating the steel target is shown. Figure 9 compares the penetration characteristics of the jet with different densities on the steel target. It can be seen from Figure 8 that the hole morphology characteristics of the jet formed by different density liners penetrating the steel target are different. When the density is 3 g/cm^3^, the diameter of the bottom of the penetrating hole is obviously smaller than that of the surface of the steel target, and the bottom of the hole is curved, and the peak pressure point inside the steel target is concentrated at the bottom of the hole. When the density is 3.5 g/cm^3^, the differentials between the diameter of the hole bottom and the diameter of the hole is reduced, and there is an obvious pressure peak point at the bottom of the hole. When the density is 4 g/cm^3^, the shape of the bottom of the hole is sharp, forming an obvious stress concentration phenomenon. Combined with Figure 9, it can be seen that the penetration depth of the steel target decreases with the increase in density, and the maximum value is 24.83 mm. At this time, the jet density is 3 g/cm^3^. The penetration aperture decreases first and then increases, and the maximum value is 18.05 mm. At this time, the jet density is 3 g/cm^3^. This is because when the jet density is 3.5 g/cm^3^, the jet head accumulates, the degree of divergence is small, and the fracture is obvious. The continuity of the jet and the expansion of the head are worse than the jet formed when the density of the liner is 3 g/cm^3^ and 4 g/cm^3^, so the penetration aperture is the smallest.

**Figure 10:**
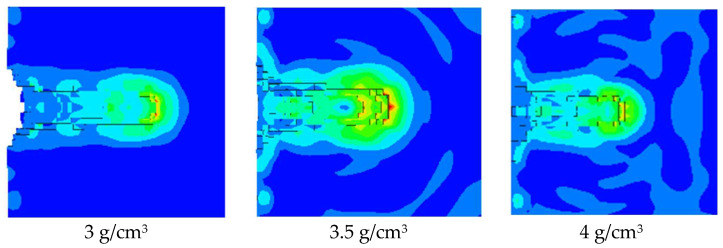
Damage effect of molded sintering PTFE/Cu jet on steel target.

**Figure 11:**
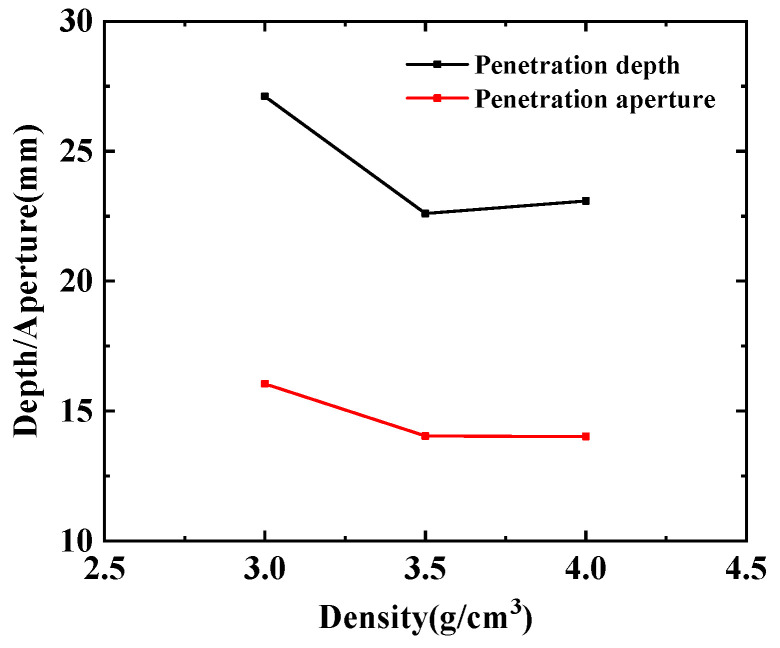
Penetration characteristics of molded sintering PTFE/Cu jet on steel target.

As shown in Figure 10, the pressure nephogram of the jet formed by the molded sintering PTFE/Cu liner penetrating the steel target is shown. Figure 11 compares the penetration characteristics of jets with different densities on the steel target. From Figure 10, it can be seen that the hole morphology characteristics formed by the penetration of different densities of molded sintering jets into the steel target are different. When the density is 3 g/cm^3^, the pressure peak of the target plate is at the bottom of the penetration hole. When the density is 3.5 g/cm^3^, the bottom of the penetrating hole gradually transits from arc to straight line, and the peak pressure is circular arc. When the density is 4 g/cm^3^, there is an aperture differential between the bottom diameter of the penetrating hole and the surface of the target plate, and there is a stress concentration point at the bottom of the penetrating hole. As shown in Figure 11, as the density increases, the depth of the jet penetrating the steel target gradually increases. When the jet density is 4 g/cm^3^, the maximum penetration depth is 27.09 mm, and the penetration aperture decreases with the increase in density. That is to say, the increase in density improves the penetration ability of the jet to the target plate and weakens the reaming effect. The main reason is that the mass ratio of Cu is more than half of the total mass, and the forming characteristics of the jet are closer to the traditional metal jet.

#### 5.1.2. Effect of Preparation Process on Jet Penetration Performance

In order to study the effect of the preparation process of the liner on the penetration performance of the jet, three sets of liners with the same density were selected for analysis, which were hot-pressing sintering, molded sintering, and extrusion molding liners with a density of 3.5 g/cm^3^. The penetration results of the steel target were compared and analyzed.

**Figure 12:**
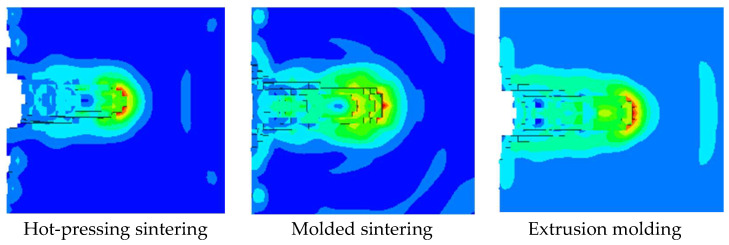
Damage effect of PTFE/Cu jet on steel target.

As shown in Figure 12, the penetration effect of the jet formed by different preparation processes on the steel target is shown. It can be seen that the different preparation processes of the liner lead to different stress concentration points when the jet penetrates the target plate. Among them, the stress concentration of molded sintering is more serious. In other words, the extrusion of the jet formed by molding sintering to the target plate is more serious, followed by hot-pressing sintering, and finally extrusion molding. According to the results of PTFE/Cu jet penetrating steel target with different processes in Table 5, it can be seen that the penetration depth of the extrusion molding liner penetrating steel target is also the largest, which is 26.5 mm, and the jet penetration aperture value is also the largest among the three liners, which is 20.16 mm. This is because in the preparation process, the sintering process of the molding sintering liner and the hot-pressing sintering liner produce new materials, and more new materials are produced in the hot-pressing sintering process. Therefore, under the same mass ratio, the Cu content of these two kinds of liners is less than that of the extrusion molding liner. Therefore, the penetration depth of the jet formed by the hot-pressing sintering liner is the smallest, and the penetration depth of the jet formed by the extrusion molding liner is the largest. Similarly, the generation of new materials also affects the reaming ability of the jet. Therefore, the penetration diameter of the jet formed by the hot-pressing sintered liner is also the smallest.

### 5.2. Experiment Verification Results

#### 5.2.1. Effect of Liner Density on Jet Penetration Characteristics

**Figure 13:**
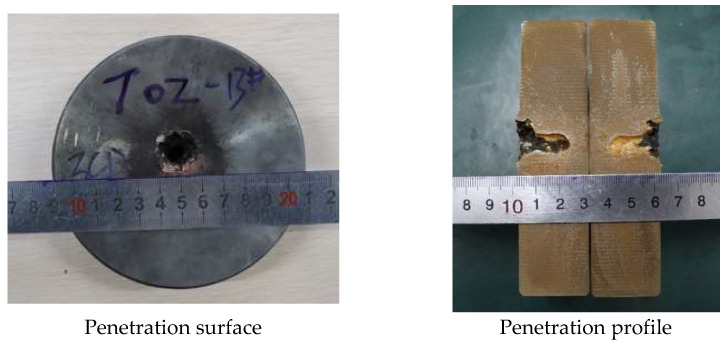
Effect diagram of PTFE/Cu jet penetrating steel target (*ρ* = 3 g/cm^3^, molded sintering).

In order to analyze the influence of liner density on jet penetration characteristics, the penetration tests of molded sintered liners with different densities were carried out. When the liner densities are 3 g/cm^3^, 3.5 g/cm^3^, and 4 g/cm^3^, the damage effect of PTFE/Cu jet penetrating steel target is shown in Figure 13.

**Figure 14:**
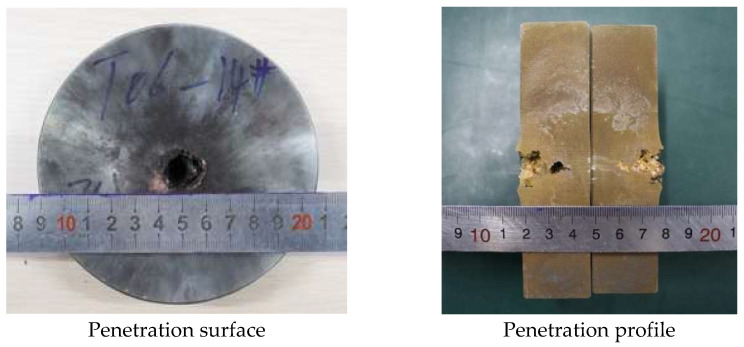
Effect diagram of PTFE/Cu jet penetrating steel target (*ρ* = 3.5 g/cm^3^, molded sintering).

**Figure 15:**
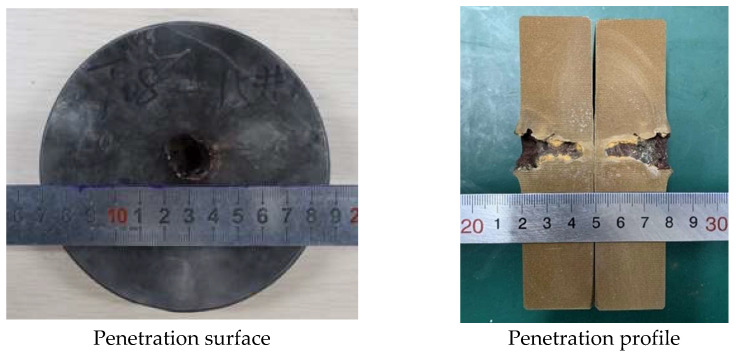
Effect diagram of PTFE/Cu jet penetrating steel target (*ρ* = 4 g/cm^3^, molded sintering).

It can be seen from the above figures that there is an obvious reaming phenomenon when the jet formed by the molded sintering liner penetrates the steel target. Observing the penetration surface, it can be seen that there is a red splash on the end face of the steel target, which is presumed to be the Cu material in the liner, and as the density increases, the splash on the surface of the steel target gradually decreases. Due to the large difference in material properties between PTFE and Cu, the particle matching degree between PTFE and Cu in the molded sintering liner is not the fit between Cu and Cu. As the density increases, the PTFE content in the liner decreases, the Cu content gradually increases, and the components that need particle matching are less. Moreover, the shape of the liner prepared by this process is regular, so the jet formation is relatively uniform and stable, and the penetration is relatively concentrated, and the offset does not occur. Therefore, the main part of the jet is used to penetrate the target plate, and the splash residue is less on the end face of the target plate. The penetration profile in Figure 13 shows that when the density of the liner is 3 g/cm^3^, the holes formed by the jet penetration are wide at the top and narrow at the bottom, and the holes at the last position of the jet head are arc-shaped. The diameter is smaller than the end face of the target plate, and the whole is “funnel-shaped”. For the penetration profile of Figure 14, it can be seen that the penetration of the jet into the steel target is uneven, and the two profiles are inconsistent. The bottom shape of one side of the jet penetration is irregular, while the other side penetrates the interior without holes. The steel target has different degrees of erosion, and the bottom position of the jet penetration is not obvious. From the penetration surface diagram, it can be seen that the jet penetrates the steel target normally when it is just in contact with the steel target. Therefore, the phenomenon may be caused by the inhomogeneity of the liner material and the poor matching of the particles. As a result, the jet cohesion is not good, and the massive objects fall one after another, resulting in the phenomenon shown in Figure 14. The jet penetration phenomenon shown in Figure 15 is similar to that in Figure 13, but the difference is that a large amount of red material can be seen in the penetration profile in Figure 15, which is the Cu component in the jet. Although the contour of the penetration hole is obvious, the contour of the hole is not as complete as that in Figure 13, and there are many edge gaps. That is to say, the increase in Cu makes the contour of the jet penetrating the steel target incomplete and blurs the penetration contour. The shape of the penetration profile under three conditions is similar to the numerical simulation results, which shows the correctness of the simulation.

**Figure 16:**
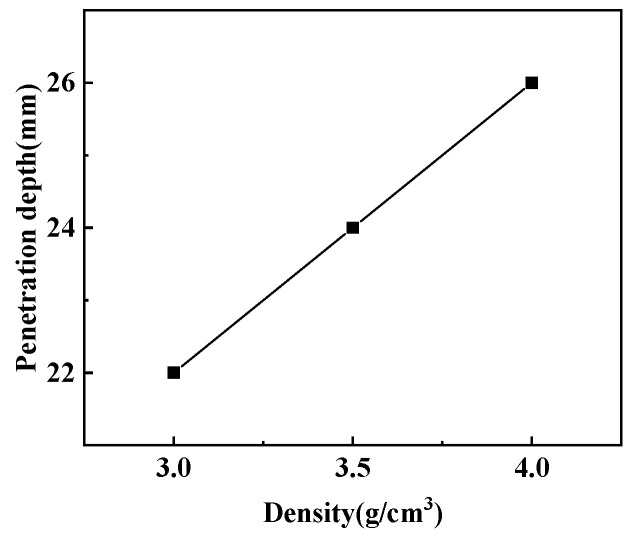
Effect of liner density on penetration depth.

**Figure 17:**
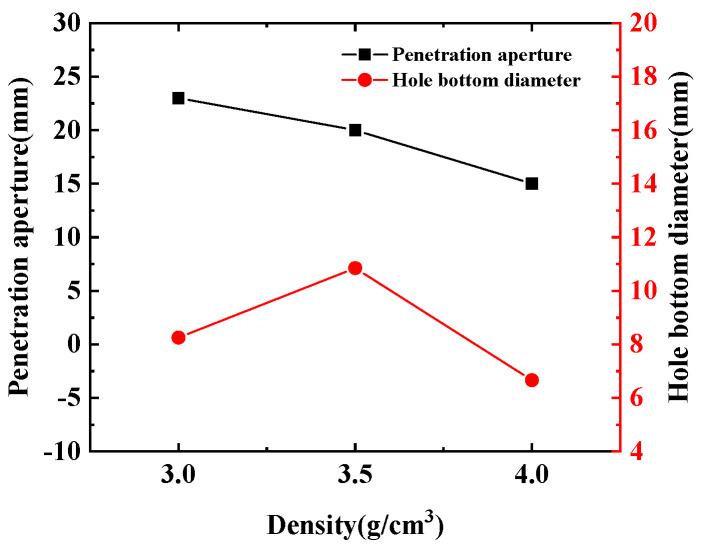
Effect of liner density on penetration aperture.

Figure 16 and Figure 17 show the effect of the density change of the molded sintering liner on the penetration depth and penetration aperture of the jet, respectively. From Figure 16, it can be seen that when the density of the liner is 3 g/cm^3^, 3.5 g/cm^3^, and 4 g/cm^3^, the penetration depth of the jet to the steel target is 22 mm, 24 mm, and 26 mm, respectively. That is to say, with the increase in density, the penetration depth of the jet also increases, and the penetration performance is enhanced. It can be seen from Figure 17 that the increase in the density of the liner is not proportional to the reaming performance of the jet. When the density is 3 g/cm^3^, the penetration aperture is 23.00 mm, and the hole bottom diameter formed by the jet penetration is 8.25 mm. When the density is 3.5 g/cm^3^, the penetration aperture is 20.00 mm, and the hole bottom diameter formed by the jet penetration is 10.85 mm. Combined with Figure 14, it can be seen that when the jet penetrates the steel target at this density, the penetration aperture is the smallest, and the inside of the penetrated hole is irregular, but the hole bottom diameter is the largest, and the penetration depth also increases with the increase in density. When the density is 4/cm^3^, the hole diameter is 15.00 mm, and the hole bottom diameter formed by jet penetration is 6.70 mm. Comparing the penetration hole diameter and hole bottom diameter with different densities, it can be seen that when the density is 3 g/cm^3^ and 4 g/cm^3^, the hole diameter difference formed by penetration is larger, and when the density of the liner is 3.5 g/cm^3^, the diameter difference is smaller than the jets of the other two densities. Comparing Figure 16 and Figure 17 with Figure 11, it is found that the penetration depth and penetration diameter of the jet in the experiment are approximately equal to the numerical simulation results, which proves the accuracy of the numerical simulation.

The numerical simulation and experimental comparison results of jet penetration depth and penetration aperture with different densities under molded sintering preparation process are shown in Table 6. Except for the penetration aperture error of 21.5% when the density is 3 g/cm^3^, the other errors are about 10% or even smaller. Therefore, it can be considered that the numerical simulation has certain accuracy.

#### 5.2.2. Effect of Preparation Process on Jet Penetration Characteristics

**Figure 18:**
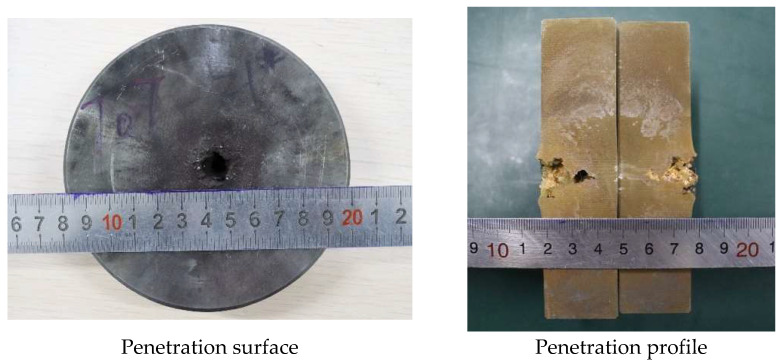
Effect diagram of PTFE/Cu jet penetrating steel target (*ρ* = 3.5 g/cm^3^, molded sintering).

**Figure 19:**
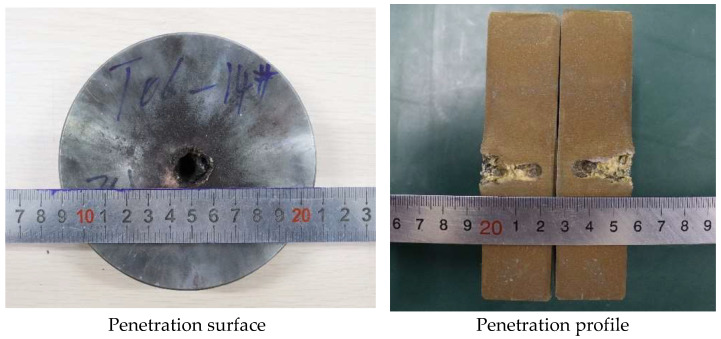
Effect diagram of PTFE/Cu jet penetrating steel target (*ρ* = 3.5 g/cm^3^, extrusion molding).

PTFE/Cu liners with a density of 3.5 g/cm^3^ were selected to compare and analyze the damage effect of the jet penetrating the steel target. The penetration surface and penetration profile of the two jets penetrating the steel target are shown in Figure 18 and Figure 19, respectively.

From Figure 18 and Figure 19, it can be seen that the different preparation processes of the liner directly affect its penetration effect on the steel target, but both have a hole expansion effect on the steel target. Comparing the influence of the liners obtained by the two preparation processes on the penetration surface of the steel target, it can be seen that Figure 18 is the penetration effect diagram of the jet formed by the molded sintering liner on the steel target. It can be seen that the penetration of the jet to the steel target is not uniform. The penetration effect is similar to erosion, the formed hole cavity is small, and the contour is fuzzy and irregular. For the extrusion molding liner, it can be seen from Figure 19 that the penetration end face of the steel target has obvious red spatter, which is the Cu component in the jet. In addition, it can be clearly seen that there is a small block at the surface of the penetrated steel target, which is due to the jet just contacting the steel target. It is caused by the divergence of the head, and because the jet divergence phenomenon is only serious in the head, and the density is much lower than that of the conventional metal jet, the penetration strength of the steel target is inconsistent, which leads to the occurrence of this phenomenon.

The maximum penetration depth of the jet formed by the molded sintering liner to the steel target is 24.00 mm, and the maximum penetration depth of the jet formed by the extrusion molding liner to the steel target is 26.00 mm. The effect of the process on the penetration depth of the jet is consistent with the effect of the penetration aperture, but the influence on the hole bottom diameter of the reaming is the opposite. The maximum penetration aperture of the molded sintering liner jet is 20 mm, and the minimum diameter of the hole bottom is 10.85 mm. The maximum penetration aperture generated by the extrusion molding liner is 20.00 mm, and the minimum diameter of the hole bottom is 7.00 mm. That is to say, the process that makes the jet penetration depth large, the diameter of the hole bottom of the penetration hole is small. This is consistent with the data results obtained by numerical simulation (Table 5).

Table 7 show the comparison of numerical simulation and experimental results under different preparation processes. It can be seen from the data in the table that the numerical simulation results are not much different from the experimental data, which verifies the accuracy of the numerical simulation.

Taking the penetration aperture of the jet formed by the extrusion molding charge liner with a density of 3.5 g/cm^3^ as an example, during the penetration process, the target strength is 790 MPa, the stagnation point pressure is 805.72 MPa, and the head diameter of the jet is 25.78 mm. Therefore, according to Equation (7), the maximum penetration diameter of the jet can be calculated to be 26.035 mm, which is very close to the test result of 26 mm. In the same way, the penetration aperture of the liner under other preparation conditions can be calculated, and then the accuracy of the hole expansion theory in Section 3 is verified.

#### 5.2.3. Effect of Sintering Sequence on Jet Penetration Characteristics

When the liner is prepared by hot-pressing sintering and molded sintering, although there is a step of sintering, the order of sintering and pressing and whether the sintering process is independent are directly related to the material properties of the prepared sample. Based on this, this section compares and analyzes the jet penetration effect of the hot-pressing sintered liner and the molded sintering liner with a density of 4 g/cm^3^. In order to better study the influence of different sintering sequence, the velocity of two kinds of jet penetrating steel target is measured.

**Figure 20:**
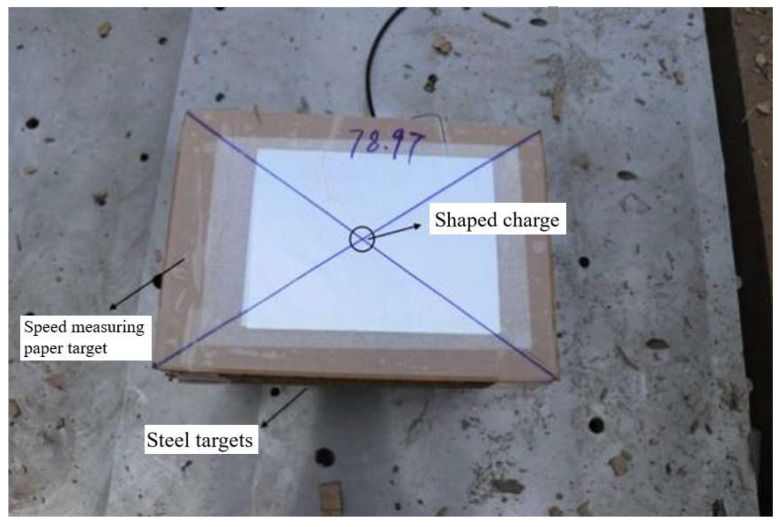
Jet velocity measuring device.

As shown in Figure 20, the velocity measuring device is set up when the jet penetrates the steel target. The velocity measuring device is placed on the steel target, and the height is twice the stand-off. The device is mainly composed of a hard paper target with an on–off resistance paper. In order to reduce the error of the jet before contacting the steel target as much as possible, the center of the paper target is hollowed out in a certain range, so that the jet is formed before penetration without any additional resistance. The principle of velocity measurement of the device is timing method. When the jet does not reach the paper target, the circuit is disconnected. When the jet reaches the paper target and penetrates the on–off resistance of the upper surface of the paper target, the circuit is connected, and the timer starts timing. Then, when the jet passes through the paper target and reaches the on–off resistance of the lower surface of the paper target, the circuit is connected again, and the timing ends. The timer shows the time when the jet head passes through the paper target. According to the velocity formula, the average velocity of the jet head through this distance is determined.

**Figure 21:**
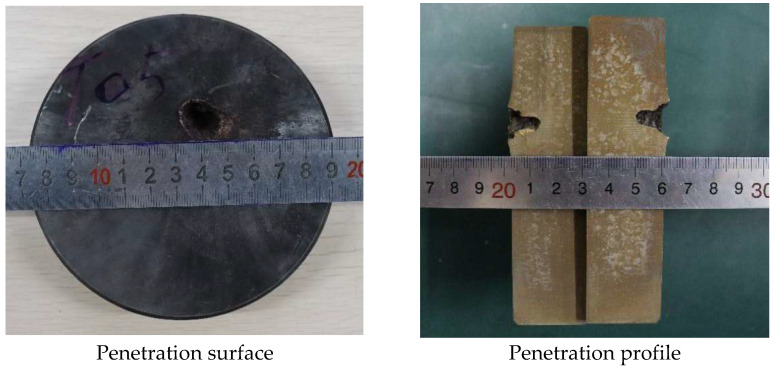
Effect diagram of PTFE/Cu jet penetrating steel target (*ρ* = 4 g/cm^3^, hot-pressing sintering).

**Figure 22:**
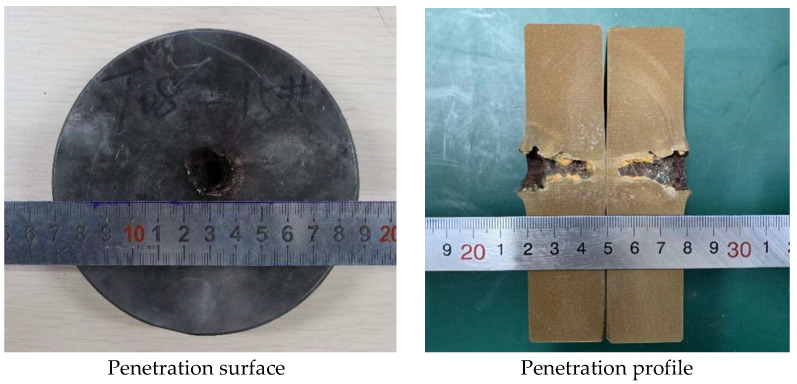
Effect diagram of PTFE/Cu jet penetrating steel target (*ρ* = 4 g/cm^3^, hot-pressing sintering).

The effects of jet penetration of steel targets formed by hot-pressing sintering and molded sintering liners with a density of 4 g/cm^3^ are shown in Figure 21 and Figure 22, respectively.

It can be seen from Figure 21 that the jet formed by the hot-pressing sintering liner is not centered when penetrating the steel target, and the penetration surface is elliptical, and some edges are everted. This is due to the extrusion during jet penetration. There are red splashing substances around the hole, which are the Cu components in the jet. It can be seen from its penetration profile that there is also an obvious hole expansion effect during the penetration of the liner under this process, and the bottom of the hole is arc-shaped rather than pointed. The contour of the penetrated hole is complete and clear, and a large number of black and red substances are attached to the inner wall of the hole. Considering the high temperature and high pressure at the moment of explosion, in addition to Cu, it may also be a Cu-based oxide. It can be seen from Figure 22 that the penetration depth of the jet formed by the molded sintering liner to the target plate is obviously greater than that of the hot-pressing sintering jet. In addition, the penetration surface of the jet formed by the molded sintering liner to the steel target is approximately circular, and there is also obvious Cu composition around the hole, and the edge of the hole accumulates and everts seriously. In other words, compared with hot-pressing sintering, the jet formed by the molded sintering liner penetrates the steel target more violently. It can be seen from the penetration profile that there are also many substances with Cu as the matrix on the inner wall of the hole. The contour of the hole is incomplete, there are a few gaps, and the closer to the bottom of the hole. The transverse and longitudinal apertures of the aperture are inconsistent, that is, the thickness and width of the jet are inconsistent during penetration. 

Table 8 shows the penetration performance parameters of the two types of liners against steel targets. It can be seen from the table that the jet formed by the hot-pressing sintering liner has a jet head velocity of 4642.86 m/s at twice the stand-off, while at the same position, the jet head velocity of the molded sintering liner is 5032.26 m/s. The velocity of the jet reaching the steel target directly affects its penetration performance. The penetration depth of the jet formed by the hot-pressing sintering liner is 13.00 mm, while the penetration depth of the molded sintering liner is 26.00 mm. The maximum diameter of the former is 19.00 mm and the minimum diameter is 15.00 mm. The hole bottom diameter is 8.33 mm, the maximum diameter of the latter is 20.00 mm, the minimum diameter is 15.00 mm, and the hole bottom diameter is 7.00 mm. Combined with the analysis results of Figure 21 and Figure 22, it can be seen that the change in sintering sequence affects the head velocity of the jet, which leads to a great difference in the penetration performance of the steel target, but has little effect on the performance of the reaming. That is to say, the size of the reaming during the penetration of PTFE/Cu jet has nothing to do with the penetration speed of the jet, which further confirms that this is due to the material characteristics of the PTFE material itself. In addition, the penetration performance of the jet formed by hot-pressing sintering liner is far less than that of molded sintering, which may also be caused by density. Considering the difference between the two preparation processes of PTFE/Cu liner, the actual density of the molded sintering liner is close to the theoretical density, while the density of the hot-pressing sintering liner is far from the theoretical density, so the jet penetration performance formed by molded sintering liner is better than that formed by the hot-pressing sintering liner. In other words, the mechanical properties of the material are improved when the sintering and pressing are carried out simultaneously during the processing of the liner, but the penetration performance of the jet is reduced. Although the mechanical properties of the sample obtained by pressing first and then sintering are not as good as the former, the penetration performance of the jet is greatly improved, and the reaming effect is not much different from the former.

Compared with Reference [19], our study considers the influence of the preparation process. In the existing literature, the preparation process of PTFE/Cu composites generally chooses cold-pressing sintering. In our study, three different processes were used to prepare the liner to compare the penetration characteristics of the jet, which provided theoretical support for the selection of the preparation method of the liner in the future. However, this paper only considers the influence of three different densities under three preparation processes. Due to the limitation of experimental conditions, the density selected in this paper is less, and the research results have certain limitations. In the future, the density range will be expanded and the density difference will be reduced for more in-depth research.

## 6. Conclusions

In this paper, the damage characteristics of PTFE/Cu jets with different preparation processes to steel targets are studied. The main conclusions are as follows:

(1) By analyzing the influence of the density change in the molded sintering liner on the penetration depth and penetration aperture of the jet, it can be seen that with the increase in density, the penetration depth of the jet also increases, and the penetration performance is enhanced. Comparing the penetration aperture and hole bottom diameter of different densities, it can be seen that when the density is 3 g/cm^3^ and 4 g/cm^3^, the hole diameter differential formed by penetration is larger, and when the density of the liner is 3.5 g/cm^3^, the aperture difference is smaller than the other two densities;

(2) PTFE/Cu jet has obvious reaming phenomenon when penetrating the steel target. The penetration characteristics of three kinds of liners with density of 3.5 g/cm^3^ are analyzed. It can be seen that due to the new material produced in the sintering process, the penetration depth and penetration aperture of the jet formed by the hot-pressing sintering liner are the smallest, followed by the molded sintering liner. The maximum penetration depth and aperture are for the jet formed by the extrusion molding liner;

(3) The mechanical properties of the material are improved when the sintering and pressing are carried out simultaneously during the processing of the liner. The penetration depth is not as good as the penetration performance of the shaped charge liner obtained by pressing first and then sintering, but the reaming effect of the two on the steel target is not much different.

## Figures and Tables

**Table 1:** Shock equation of state parameters and Johnson–Cook parameters of PTFE/Cu material.

Liner No.	Preparation Process	ρ (g/cm^3^)	Gruneisen Coefficient	C1	S1	Yield Strength (MPa)	Hardening Constant (MPa)
1#	Hot-pressing sintering	3	2.7482	2.1433	1.7045	17.13	32.726
2#		3.5	2.7915	1.9671	2.2286	20.31	34.507
3#		4	2.8073	2.0327	1.7881	19.47	46.680
4#	Molded sintering	3	2.7482	2.1662	1.7472	12.16	40.083
5#		3.5	2.7915	2.1218	1.7064	16.72	48.122
6#		4	2.8073	2.0732	1.8137	20.396	60.039
7#	Extrusion molding	3	2.7482	2.1175	1.7329	14.35	16.306
8#		3.5	2.7915	2.1792	1.1618	9.73	41.897

**Table 2:** JWL equation of state parameters and C-J parameters of explosive 8701.

*A* (GPa)	*B* (GPa)	*R*_1_	*R*_2_	*W*	*ρ* (g/cm^3^)	*D* (m/s)	*E* (KJ/m^3^)	*P_CJ_* (GPa)
854.5	20.493	4.6	1.35	0.25	1.71	8315	8.5e6	29.5

**Table 3:** Parameters of 4340 steel.

Material	ρ (g/cm^3^)	*A* (Mba)	*B*	*n*	*C*	*m*
Steel 4340	7.83	0.0079	0.0051	0.26	0.014	1.03

**Table 4:** Penetration test parameters.

Liner No.	Preparation Process	Density (g/cm^3^)	Stand-Off
1#	Hot-pressing sintering	3	3D
2#		4	2D
3#	Molded sintering	3	3D
4#		3.5	3D
5#		4	2D
6#	Extrusion molding	3.5	3D

**Table 5:** Experimental parameters of PTFE/Cu jet penetrating steel target.

Liner Material	Penetration Aperture (mm)	Penetration Depth (mm)
Hot-pressing sintering	12.08	21.6
Molded sintering	17.85	24.6
Extrusion molding	20.16	26.5

**Table 6:** Comparison of the results under the different densities of molded sintering liners.

Density (g/cm^3^)	Experimental Condition	Penetration Depth (mm)	Penetration Aperture (mm)
3	Numerical simulation	24.83	18.05
	Experiment verification	22	23
	Error	12.8%	21.5%
3.5	Numerical simulation	24.6	17.85
	Experiment verification	24	20
	Error	2.5%	10.75%
4	Numerical simulation	27.09	14.02
	Experiment verification	26	15
	Error	4.19%	6.5%

**Table 7:** Comparison of the results under the different preparation processes at 3.5 g/cm^3^.

Preparation Process	Experimental Condition	Penetration Depth (mm)	Penetration Aperture (mm)
Molded sintering	Numerical simulation	24.6	17.85
	Experiment verification	24	20
	Error	2.5%	10.75%
Extrusion molding	Numerical simulation	26.5	20.16
	Experiment verification	26	20
	Error	1.92%	0.8%

**Table 8:** Penetration performance parameters of jet.

Liner Type	Density (g/cm^3^)	Velocity (m/s)	Penetration Depth (mm)	Penetration Aperture (mm)	Hole Bottom Diameter (mm)
Hot-pressing sintering	4	4642.86	13	19*15	8.33
Molded sintering	4	5032.26	26	20*15	7.00

## Data Availability

Not applicable.
